# Accurately assembling nanopore sequencing data of highly pathogenic bacteria

**DOI:** 10.1186/s12864-025-11793-6

**Published:** 2025-08-28

**Authors:** Christine Thomas, Hanka Brangsch, Valentina Galeone, Martin Hölzer, Manja Marz, Jörg Linde

**Affiliations:** 1 Institute of Bacterial Infections and Zoonoses, Federal Research Institute for Animal Health, Friedrich-Loeffler-Institute, Naumburger Str. 96a, 07743 Jena, Germany; 2 RNA Bioinformatics and High-Throughput Analysis, Friedrich Schiller University Jena, Leutragraben 1, 07743 Jena, Germany; 3 Bioinformatics and Translational Research, Genome Competence Center, Robert Koch Institute, Nordufer 2, 13353 Berlin, Germany; 4 BIC - Bioinformatics Core Facility, Friedrich Schiller University Jena, Leutragraben 1, 07743 Jena, Germany

**Keywords:** Oxford nanopore technologies, Assembly pipeline, Bacteria, Pathogens, Outbreak analysis

## Abstract

**Background:**

Bacterial genome exploration and outbreak analysis rely heavily on robust whole-genome sequencing and bioinformatics analysis. Widely-used genomic methods, such as genotyping and detection of genetic markers demand high sequencing accuracy and precise genome assembly for reliable results.

**Methods:**

To assess the utility of nanopore sequencing for genotyping highly pathogenic bacteria with low mutation rates, we sequenced six reference strains using Oxford Nanopore Technologies (ONT) R10.4.1 chemistry and Illumina and evaluated different assembly strategies. The publicly available RefSeq assemblies were chosen as the ground truth. Publicly available sequencing data from key foodborne and public-health-related bacterial pathogens were examined to provide a broader context for the analysis.

**Results:**

While for *Bacillus* (*Ba*.) *anthracis* an almost perfect assembly was achieved, results varied for other species. For *Brucella* (*Br*.) spp., the final assemblies comprised five to 46 different nucleotides in comparison to Sanger-sequenced references. For some key foodborne and public-health-related bacterial pathogens (*Klebsiella* (*K.*) *variicola*, *Listeria* spp., *Mycobacterium* (*M.*) *tuberculosis*, *Staphylococcus* (*Sta.*) *aureus*, and *Streptococcus* (*Str.*) *pyogenes*) perfect genomes were obtained. Enhanced basecalling models have generally improved assembly accuracy, however, for certain species such as *Br. abortus*, older models have produced higher accuracy. While long-read polishing mainly improves assembly quality with only one round needed, our results indicate that this process may also degrade assembly quality. Overall, 81% of the observed errors in ONT assemblies were located within coding sequences (CDS). Furthermore, we found that methylation caused 6.5% of the errors, and the bacterial methylation-aware medaka polishing model reduced the number of errors linked to methylation. Core-genome Multilocus Sequence Typing (cgMLST) analysis revealed allele differences in *Ba. anthracis, Br. abortus*, and *Francisella (F.) tularensis* for some assemblers, although with fewer than five allele differences. In the case of *Br. melitensis*, some assemblies included five allele differences, whereas for *Br. suis* the correct cgMLST alleles were observed.

**Conclusions:**

Assembling nanopore data from pathogenic bacteria vary in quality across different species and methods. However, errors persist in the final assemblies, including within cgMLST loci, influencing the reliability of outbreak predictions. Nevertheless, specific combinations of existing tools can generate perfect genome assemblies from bacterial ONT sequencing data for outbreak analysis without short-read polishing.

**Supplementary Information:**

The online version contains supplementary material available at 10.1186/s12864-025-11793-6.

## Background

Next-generation sequencing (NGS) has reshaped the field of microbial genomics by facilitating the high-throughput reconstruction of bacterial genomes for diagnostics. Bioinformatics methods have been developed at an equal pace, enhancing the reliability of the analysis of sequencing data. Especially, highly scalable and parallelized computational pipelines ease the analysis of comprehensive data sets.

The nanopore sequencing technology, with commercially available devices provided by Oxford Nanopore Technologies (ONT), is increasingly applied in microbial genomics [1]. Its ability to generate ultra-long reads significantly improves the assembly process, achieving greater continuity and potentially resulting in closed de novo genome reconstructions [2]. Low acquisition costs and simplified sample preparation processes are additional advantages, particularly for small laboratories with limited resources. Applications ranging from telomere-to-telomere reconstruction [3] to genetic markers, plasmid investigations [4, 5], and extending to metagenomics studies [6], underscore the advantages of nanopore sequencing. However, technology-specific systematic error patterns [7, 8], in combination with frequent changes in product lines and quality fluctuations [9], continue to pose significant challenges for downstream bioinformatics analyses and toolchains. Hence, hybrid assembly methods, which combine long-read and short-read sequencing technologies, are still considered the gold standard [2]. Nevertheless, these combined techniques are often not feasible for smaller laboratories due to their high acquisition costs. Although Illumina short-read sequencing remains the dominant sequencing method [10, 11], nanopore sequencing is gaining traction in bacterial epidemiology because of its rapid sequencing capabilities, which enable real-time monitoring and identification of pathogens [12–14].

Effective outbreak analysis often requires nucleotide-level precision, necessitating highly accurate sequencing and genome reconstruction. This is particularly important for bacteria with low mutation rates, where even a small number of genomic differences can be crucial distinguishing between outbreaks [15, 16]. Compared with traditional approaches such as multilocus sequence typing (MLST) and multiple-locus variable number of tandem repeats analysis (MLVA), whole-genome sequencing (WGS) facilitates higher resolution genotyping methods, such as core-genome Multilocus Sequence Typing (cgMLST) and single nucleotide polymorphism (SNP) typing [10, 17–19]. ONT shows promise in outbreak analysis for some bacteria [20], in terms of molecular genotyping and phylogenetic analysis [21–23], plasmid characterization [24], and species identification via 16S rDNA sequencing [25]. Nonetheless, it has led to significant issues for other species [15, 23, 26–28]. One potential reason for this finding involves issues with DNA methylation bases during the basecalling process (where the measured electrical signal is translated to the nucleotide) in ONT sequencing data from bacteria [26–30]. Less studied bacterial species could present a particular challenge, as they may be underrepresented in the test and training datasets used by various bioinformatic methods, which may influence their accuracy [31].

In this study, the accuracy of assemblies obtained with different assembling strategies was evaluated for highly pathogenic bacteria. To this end, six highly pathogenic bacteria with rather low mutation rates (*Bacillus* (*Ba*.) *anthracis, Brucella* (*Br*.) *abortus, Brucella* (*Br*.) *suis, Brucella* (*Br*.) *melitensis, Francisella* (*F.*) *tularensis*, and *Taylorella* (*T.*) *equigenitalis)* were sequenced, and the Sanger-sequenced reference genomes obtained from NCBI were used for evaluation. To assess mutations caused by recultivation, the same DNA also was sequenced with Illumina. Additionally, publicly available data were integrated to test the strategies across a broader spectrum of bacterial species and assess the differences between well studied and less studied species. The quality and error profiles of long-read assemblies were investigated and cgMLST analysis was performed. A pipeline was developed to facilitate automated high-quality bacterial genome reconstruction from raw sequencing data.

## Results

### Sequencing results and genomic characteristics

Six reference strains of highly pathogenic bacteria with low mutation rates were sequenced with ONT and Illumina in this study (Tables 1 and 2). These strains are denoted as the *selection group*. Additionally, the *comparison group* contains a spectrum of bacteria with high importance in the medical and food safety sectors to frame the results within a broader perspective (Tables 1 and 2). The data were published by Hall et al. [32]. While the species differed in genome size, GC content, and the number of chromosomes/plasmids, the raw sequencing data also displayed variations in quality (Tables 1 and 2).

**Table 1:** Genomic characteristics of the *selection group* and the *comparison group (Mbp - million base pairs)*

Strain	Genome size [Mbp]	Chromosomes/plasmids	GC content
***Selection group***			
Ba. anthracis Ames Ancestor	5.5	1 chromosome, 2 plasmids	35.25
Br. abortus bv. 2 86/8/59	3.3	2 chromosomes	57.22
Br. melitensis 16M	3.3	2 chromosomes	57.21
Br. suis ATCC 23445	3.3	2 chromosomes	57.19
F. tularensis LVS	1.9	1 chromosome	32.15
T. equigenitalis ATCC 35865	1.7	1 chromosome	37.37
***Comparison group***			
C. jejuni ATCC-33560	1.7	1 chromosome	30.22
C. lari ATCC-35221	1.5	1 chromosome	29.81
E. coli ATCC-25922	5.2	1 chromosome, 4 plasmids	50.37
K. pneumoniae KPC2	5.6	1 chromosome, 3 plasmids	56.89
K. variicola AJ292	5.4	1 chromosome	57.62
L. ivanovii ATCC-19119	2.9	1 chromosome	37.15
L. monocytogenes ATCC-BAA-679	2.9	1 chromosome	37.98
L. welshimeri ATCC-35897	2.8	1 chromosome	36.35
M. tuberculosis mc26030	4.4	1 chromosome	65.62
Sa. enterica ATCC-10708	4.8	1 chromosome, 1 plasmid	52.17
Sta. aureus BPH2947	3	1 chromosome, 2 plasmids	32.79
Str. dysgalactiae STG643	2.2	1 chromosome	39.49
Str. pyogenes BPH2947	1.7	1 chromosome	38.32
V. parahaemolyticus ATCC-17802	5.1	2 chromosomes	45.32

**Table 2:** Sequencing metrics of the *selection group* and the *comparison group* (bp—basepairs)

Strain	Median read length [bp]	Mean read length [bp]	Mean quality	% of reads > Q15	Estimated coverage
***Selection group***					
Ba. anthracis Ames Ancestor	2133	3537	16.5	85.1	57
Br. abortus bv. 2 86/8/59	2687	3440.5	15.2	78	151
Br. melitensis 16M	3192	4105.1	14.8	75.4	151
Br. suis ATCC 23445	351	648.7	17.5	85.1	151
F. tularensis LVS	1425.5	3616.4	15.8	82.9	65
T. equigenitalis ATCC 35865	469	807.7	17.5	88.9	294
***Comparison group***					
C. jejuni ATCC-33560	1521	3450.6	14.9	77.8	278
C. lari ATCC-35221	1717	3702.5	14.8	78.1	325
E. coli ATCC-25922	724	1435.9	15.1	80	84
K. pneumoniae KPC2	3963	9355.4	15	77.2	13
K. variicola AJ292	3147	8237.2	18.1	90.4	89
L. ivanovii ATCC-19119	1285	2922.4	14.7	76	166
L. monocytogenes ATCC-BAA-679	1400	2983.9	14.5	74.9	161
L. welshimeri ATCC-35897	1963	3938.9	15.6	81.6	174
M. tuberculosis mc26030	3227	3856.6	18.1	91.9	112
Sa. enterica ATCC-10708	610	1185.9	17.8	89.8	70
Sta. aureus BPH2947	1276	3974.1	17.8	89.8	18
Str. dysgalactiae STG643	1990	4084.2	16.1	85.1	227
Str. pyogenes BPH2947	1539	2897.9	15.6	82.2	280
V. parahaemolyticus ATCC-17802	652	1396	14.9	76.8	79

*Ba. anthracis* had the largest genome of 5.5 Mbp, consisting of one chromosome and two plasmids, followed by *Brucella* spp., all with 3.3 Mbp and two chromosomes. The smallest genome was that of *T. equigenitalis* with 1.7 Mbp and one chromosome. The *F. tularensis* genome consisted of one chromosome and had a slightly larger genome size of 1.9 Mbp. Furthermore *T. eguigenitalis*, *Ba. anthracis* and *F. tularensis* had low GC contents of 32.15%, 35.25%, and 37.37%, respectively, whereas *Brucella* spp. had a GC content of approximately 57%.

*Br. melitensis* sequencing produced long reads, with a median of 3192 bp and an average of 4105 bp, whereas the *Br. suis* data had shorter read lengths (median of 351 bp and average of 648.7 bp). In terms of high-quality reads (Q15), *Br. suis* and *T. equigenitalis* performed well, with 88.1% and 88.9% of reads, respectively, whereas *Br. melitensis* reads had a slightly lower quality, with 75.4% reaching Q15. Coverage additionally varies; sequencing data from *T. equigenitalis* had the highest estimated coverage at 285X, while sequencing data from *F. tularensis* had the lowest coverage at 70X.

Within the *comparison group* (Table 1), *Klebsiella* (*K.) pneumoniae* had the largest genome with 5.6 Mbp consisting of one chromosome and three plasmids. The *Escherichia* (*E.) coli* reference genome included the largest number of plasmids, i.e. four, with a total genome size of 5.2 Mbp*. Campylobacter (C.) jejuni* had the smallest genome size of the strains with 1.7 Mbp. Most of the strains within the comparison group had a GC content of around 30–40%, except for *E. coli*, *Klebsiella* spp., *Salmonella (Sa.) enterica*, and *Vibrio (V.) parahaemolyticus,* which had 45–60%. The highest GC content had *Mycobacterium (M.) tuberculosis* with 65.62%.

In the *comparison group*, the average read length varied between 1185 bps (*Sa. enterica*) and 9355 (*K. pneumoniae*). Despite the low coverage of 13X, *K. pneumoniae* resulted in the longest reads on average. The next lowest coverage was observed for *Staphylococcus (Sta.) aureus* (18X), which is considerably low for accurate genome assembly. The percentage of reads reaching Q15 and higher varied between 74.9% (*L. monocytogenes*) and 91.9% (*M. tuberculosis*).

### Which assembly approach results in the most accurate assembly per species?

#### Approach and measurements

**Fig. 1:**
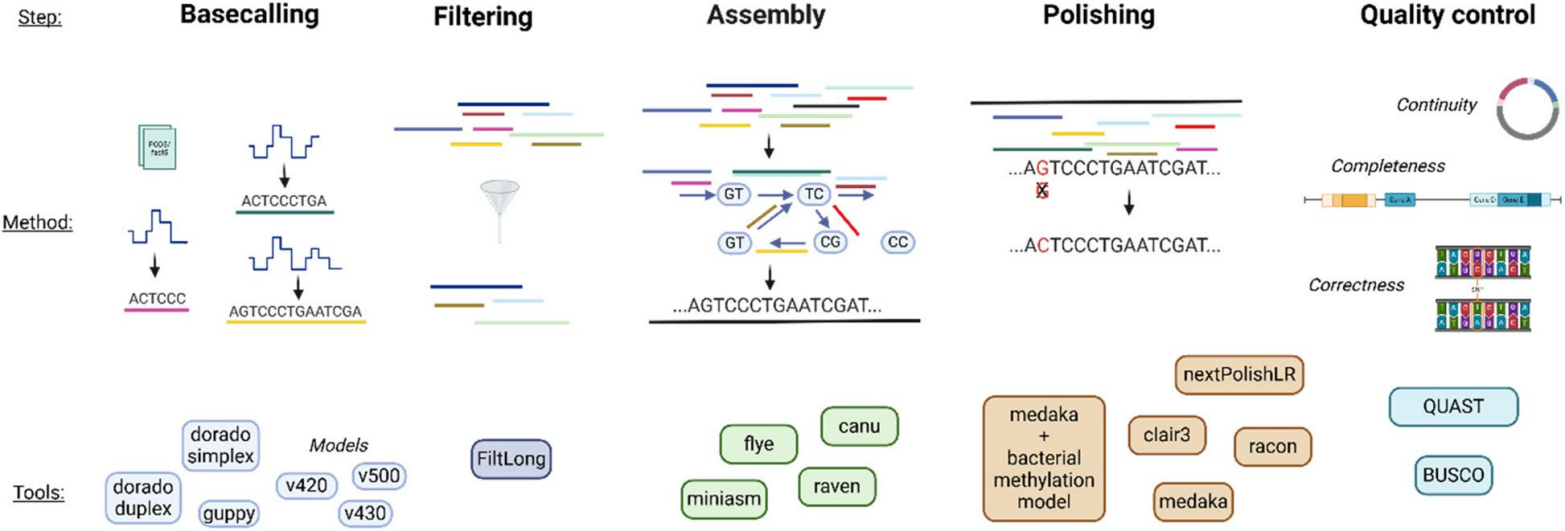
Tools selected for evaluation for each step of genome reconstruction (basecalling, assembly, polishing). Created with BioRender

In outbreak analysis, choosing accurate sequencing technology and follow-up bioinformatics is crucial for obtaining reliable, actionable results. Evaluating assembly strategies ensures that the technology can effectively handle complex bacterial genomes and provide high-quality data for precise analysis, including genotyping. To evaluate the available options for de novo assembly reconstruction, various combinations of basecallers, basecalling models, assemblers, and polishers (Fig. 1) were benchmarked using sequencing data from the different strains of the *selection group*. Additionally, publicly available sequencing data for common bacterial pathogens were included in the benchmarking. In this context, the *combined error rate *${E}_{combined} \left(AS(A,P,r,i)\right)$ was used to investigate the performance (see definition formula (1) in Methods).

#### Basecalling performance varies between species

Precise basecalling is the foundation of correct genome reconstruction. To date, ONT has two widely applied basecallers guppy and dorado, with dorado having two modes, simplex and duplex. These basecallers utilize recurrent neural networks, called models, for basecalling. The models are trained on specific training sets and are frequently updated to improve the basecalling process. To verify which basecaller is superior as well as whether newer models improve the performance of basecalling, we tested different combinations for all strains. For basecalling, the available ONT basecaller options guppy, dorado simplex, and dorado duplex were tested with the model version v420 (dna_r10.4.1_e8.2_400bps_sup@v4.2.0, published 18 May 2023). Additionally, with dorado duplex, the most recent basecalling models v420, v430 (dna_r10.4.1_e8.2_400bps_sup@v4.3.0, published 5 Dec 2023), v500 (dna_r10.4.1_e8.2_400bps_sup@v5.0.0, published 21 May 2024) were tested. Genome assemblies were constructed by filtering reads with Filtlong, and subsequently using ONT’s recommended assembly tool [33] flye and medaka (with corresponding medaka models dna_r10.4.1_e8.2_400bps_sup@v4.2.0, dna_r10.4.1_e8.2_400bps_sup@v4.3.0, and dna_r10.4.1_e8.2_400bps_sup@v5.0.0) for polishing. The quality was measured by the combined error ${E}_{combined}\left(AS\left(A,P,r,i\right)\right), with\ A = flye, P = medaka,$ $r = 1$ for strain $i, i\in S, S$ including the strains of the two different groups and ${\overline{E} }_{combined}$ being the average of the different groups of strains (see definition formula (1) in Methods).

**Fig. 2:**
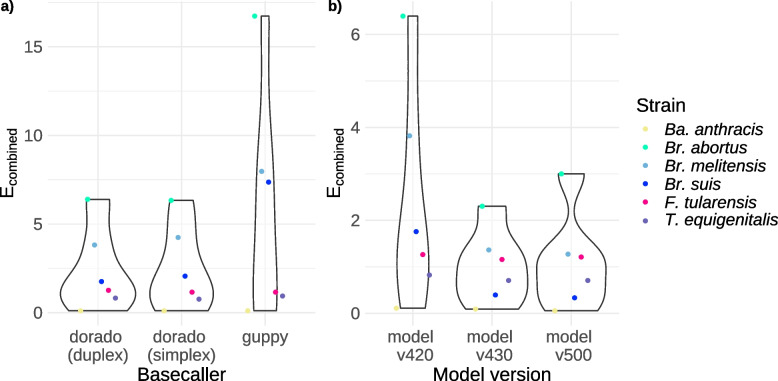
Evaluation of the basecallers and models influencing the accuracy of the final assembly (*selection group*). **a** Violin plot of ${E}_{combined}$ of the final assemblies for dorado duplex, dorado simplex, and guppy with model v420. **b** Violin plot of ${E}_{combined}$ of the final assemblies for dorado duplex using models v420, v430, and v500

Dorado in duplex mode gave the best overall performance across the *selection group* (Fig. 2a, supplement file 2: Basecalling) with a mean combined error rate ${\overline{E} }_{combined}=2.36$ compared to dorado simplex with ${\overline{E} }_{combined}=2.44$ and guppy with ${\overline{E} }_{combined}=5.71$. Only for *Br. abortus* and *T. equigenitalis*, dorado simplex was the best option with two and one fewer errors, respectively, compared to dorado duplex, and for *F. tularensis* dorado simplex and guppy resulted in two fewer errors than dorado duplex. Considering the results across the *comparison group* (Figure S1a, supplement file 2: Basecalling), dorado duplex scored best, with an average of ${\overline{E} }_{combined}=2.37$ compared to dorado simplex with ${\overline{E} }_{combined}=2.69$ and guppy with ${\overline{E} }_{combined}=2.82$. However, dorado simplex scored best for *E. coli, Sa. enterica*, and *M. tuberculosis* with 10, 13, and one error(s) fewer, respectively, than dorado duplex. For *L. welshimeri*, guppy resulted in one error less compared to both dorado modes.

Next, we investigated the performance of different dorado models. For the *selection group* (Fig. 2b, supplement file 2: Basecalling), model v420 achieved ${\overline{E} }_{combined}=2.36$, model v430 ${\overline{E} }_{combined}=1.00$ (performed best), and model v500 ${\overline{E} }_{combined}=1.09$ within the *selection group*. Results varied between species, e.g. *Br. abortus* resulted in the best assembly with model v430, even though overall an improvement of the models over time was observed.

A slightly different trend was observed when considering the *comparison group* (Figure S1b, supplement file 2: Basecalling). Each basecalling model improved on average compared with previously published basecalling model. Hence, model v420 resulted in ${\overline{E} }_{combined}=2.38$, model v430 resulted in ${\overline{E} }_{combined}=1.48$, and model v500 resulted in ${\overline{E} }_{combined}=0.72$.

#### No clear outperformance of one assembly approach across all strains

To determine whether one assembler together with a specific polishing approach outperformed other combinations, all combinations of assemblers and polishers were explored (with up to three rounds of polishing). The different assemblies were created with filtered, basecalled (dorado duplex, model v500) reads. The assembly approaches were evaluated with the combined error rate and $$AS^{*}\left(A,P,r+1,i\right)=arg\;\underset{}{min_P\;}$$$$E_{combined}\;\left(AS\left(A,P,r,i\right)\right)$$ (definition formula (2) in the Methods). All assemblies of each assembler were polished with all polishers and the assembly with the lowest $${E}_{combined}$$ was taken for the next round of polishing. If multiple strategies resulted in the minimal $${E}_{combined}$$, $$noPolishing$$ was taken (if possible), or a random choice of polisher. In total, up to three rounds of polishing were performed (Table 3; supplement file 2: Assembly).

**Table 3:** Assembly strategy with the lowest ${E}_{combined}$ for the *selection group* and* comparison group*. All possible assemblers (canu, flye, miniasm, and raven) were combined with each polisher (clair3, medaka, racon, nextPolishLR, medakaBM, or no polishing) and the assembly with the lowest ${E}_{combined}$ was taken for the next round if there was an improvement compared to the last round of polishing (up to three rounds)

Strain	Group	Assembler	Round 1	Round 2	Round 3	SNPs	Ins	Del
*Ba. anthracis*	Selection	raven	clair3	no polishing	no polishing	0	0	2
*Br. abortus*	Selection	flye	medakaBM	no polishing	no polishing	32	13	1
*Br. melitensis*	Selection	flye	nextPolishLR	no polishing	no polishing	14	18	7
*Br. melitensis*	Selection	miniasm	nextPolishLR	nextPolishLR	no polishing	14	18	7
*Br. suis*	Selection	flye	medakaBM	no polishing	no polishing	0	3	2
*F. tularensis*	Selection	flye	medaka	no polishing	no polishing	12	2	8
*F. tularensis*	Selection	miniasm	clair3	no polishing	no polishing	12	2	8
*F. tularensis*	Selection	raven	clair3	no polishing	no polishing	12	2	8
*T. equigenitalis*	Selection	flye	medakaBM	no polishing	no polishing	6	2	2
*C. jejuni*	Comparison	flye	medakaBM	no polishing	no polishing	0	0	1
*C. jejuni*	Comparison	raven	medakaBM	no polishing	no polishing	0	0	1
*C. lari*	Comparison	flye	medakaBM	medakaBM	no polishing	1	0	3
*C. lari*	Comparison	miniasm	medakaBM	no polishing	no polishing	1	0	3
*E. coli*	Comparison	flye	clair3	no polishing	no polishing	2	0	0
*K. pneumoniae*	Comparison	flye	racon	clair3/racon	no polishing	13	8	26
*K. variicola*	Comparison	flye	nextPolishLR	medakaBM	no polishing	0	0	0
*K. variicola*	Comparison	miniasm	medakaBM	no polishing	no polishing	0	0	0
*K. variicola*	Comparison	raven	medakaBM	no polishing	no polishing	0	0	0
*L. ivanovii*	Comparison	flye	medaka	no polishing	no polishing	0	0	3
*L. ivanovii*	Comparison	miniasm	medakaBM	no polishing	no polishing	0	0	3
*L. ivanovii*	Comparison	raven	medaka	no polishing	no polishing	0	0	3
*L. monocytogenes*	Comparison	flye	medakaBM	no polishing	no polishing	0	0	0
*L. monocytogenes*	Comparison	raven	clair3/nextPolishLR/medaka/medakaBM	no polishing	no polishing	0	0	0
*L. welshimeri*	Comparison	flye	medaka	no polishing	no polishing	0	0	0
*L. welshimeri*	Comparison	miniasm	medaka/medakaBM	no polishing	no polishing	0	0	0
*L. welshimeri*	Comparison	raven	nextPolishLR/medakaBM	no polishing	no polishing	0	0	0
*M. tuberculosis*	Comparison	flye	medakaBM	no polishing	no polishing	0	0	0
*Sa. enterica*	Comparison	miniasm	nextPolishLR	clair3	no polishing	0	0	6
*Sta. aureus*	Comparison	flye	medakaBM/racon	no polishing	no polishing	0	0	0
*Sta. aureus*	Comparison	miniasm	clair3	no polishing	no polishing	0	0	0
*Str. dysgalactiae*	Comparison	miniasm	medakaBM	medakaBM	no polishing	0	0	2
*Str. pyogenes*	Comparison	flye	clair3/nextPolishLR/medaka/medakaBM	no polishing	no polishing	0	0	0
*Str. pyogenes*	Comparison	miniasm	nextPolishLR	no polishing	no polishing	0	0	0
*Str. pyogenes*	Comparison	raven	clair3/nextPolishLR/medaka/medakaBM	no polishing	no polishing	0	0	0
*V. parahaemolyticus*	Comparison	flye	medakaBM	medakaBM	no polishing	1	0	0

Often, multiple assembler-polisher combinations did result in the minimum ${E}_{combined}$ (in Table 3 indicated with^1^, supplement files 2: Assembly). Additionally, especially for flye, only one round of polishing was needed as additional rounds did not result in improved assemblies (marked as “no polishing” for rounds two and three in Table 3). Overall, strains belonging to the *comparison group* did lead to more accurate assemblies than those belonging to the *selection group*. The most accurate assemblies contained zero differences. This was the case for five strains of the *comparison group*. The least accurate assembly was from *K. pneumoniae* with ${E}_{combined}=47$. Flye was most often part of the best assembly strategy (for 17 out of 20 strains). MedakaBM was most often part of the optimal strategy (for 20 out of 35 optimal strategies).

#### Flye produces the most continuous and complete assemblies

To investigate the continuity and completeness of the assemblies, for each assembler the most accurate assembly was taken for each strain according to the calculated error rate. Continuity was measured with the average deviation $\overline{Dev }(A{S}_{A})$ (definition formula (3) and (4) in Methods). Additionally, the occurrence of inversions was assessed. Completeness was assessed with BUSCO by identifying conserved single-copy orthologs.

Regarding continuity, flye created the most continuous assemblies (Figure S2, supplement 2: Contigs) with $\overline{Dev }\left(A{S}_{flye}\right)=1$ $for\ i \in {S}_{selection}$, hence on average every assembly had one contig more than the reference for strains from the *selection group*. Canu performed worst considering continuity with $\overline{Dev }(A{S}_{canu})=43.2$ $for\ i \in {S}_{selection}$. Considering the *comparison group*, flye created again the most continuous assemblies with on average $\overline{Dev }(A{S}_{flye})=0.5$ $for\ i \in {S}_{comparison}$, while again canu performed worst $\overline{Dev }(A{S}_{canu})=32.71$ $for\ i \in {S}_{comparison}$. Flye did not assemble one of the plasmids for *E. coli* (therefore, the deviation was −1). Extensive inversions were identified solely in assemblies generated by flye for *E. coli* (1 inversion) and *K. variicola* (2 inversions), as well as by canu for *E. coli* (2 inversions) and *T. equigenitalis* (7 inversions).

Of the respective universal single copy orthologs (BUSCOs) for each species (supplement files 2: BUSCO), flye detected on average 98.47% as complete and single copy, whereas canu detected the lowest fraction (86.84% on average) complete and single copy BUSCOs. Complete and duplicated BUSCOs were present most in canu assemblies with 9.48% on average, and least abundant in miniasm assemblies with 0.2% on average. Canu assemblies additionally included the most fragmented BUSCOs with on average 1.45%, the other assemblers resulted on average on 0.25–0.28% of fragmented BUSCOs. Canu assembler resulted in 2.23% missing BUSCOs, while the other assemblies led to on average 1.04%—1.51% of missing BUSCOs.

### What is the best assembly strategy across strains and what is causing the errors?

#### Flye statistically outperformed other assemblers

**Fig. 3:**
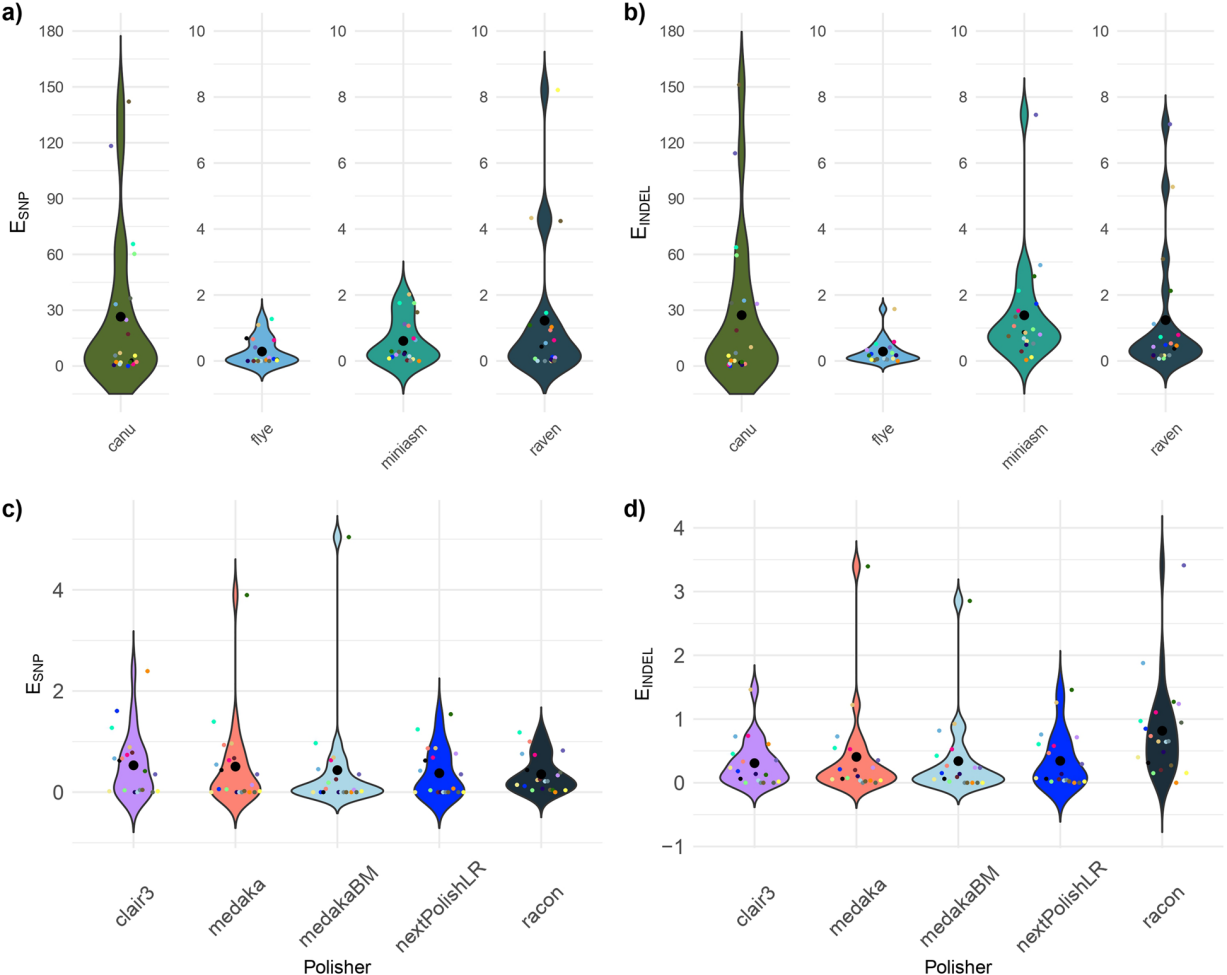
Violin plots showing the distributions of ${E}_{SNP}$ and ${E}_{INDEL}$ by the assembler and polisher. **a** Violin plot for ${E}_{SNP}$ for the different assemblers without polishing. **b** Violin plot for ${E}_{INDEL}$ for the different assemblers without polishing **c**) Violin plot for ${E}_{SNP}$ for the different polishers with flye assemblies. d Violin plot for ${E}_{INDEL}$ for the different polishers with flye assemblies. The colored smaller points represent ${E}_{SNP}$ and ${E}_{INDEL}$, respectively. The larger black dots represent the means ${\overline{E} }_{SNP}$ and ${\overline{E} }_{INDEL}$, respectively

To test the performance of the assemblers, ${E}_{combined}$, ${E}_{SNP}$, and ${E}_{INDEL}$ (definition of formula in Methods) before polishing $(P=noPolishing)$ were evaluated (Fig. 3a, b). There was no statistically significant difference globally across all assemblers for ${E}_{SNP}$ considering all strains. Nevertheless, pairwise differences could be identified: canu performed worse compared to flye (Z = 5.29, *p* < 0.05), miniasm (Z = 3.73, *p* < 0.05), and raven (Z = 3.69, *p* < 0.05). For ${E}_{INDEL}$, the Kruskal–Wallis test showed a significant difference in the error rates between the four assemblers. Canu performed significantly worse compared to all other assemblers, i.e. flye (Z = 5.81, *p* < 0.05), miniasm (Z = 2.52, *p* < 0.05), and raven (Z = 3.50, *p* < 0.05). Flye and miniasm showed a statistically significant difference in ${E}_{INDEL}$ with flye outperforming miniasm (Z = −3.33, *p* < 0.05), Fig. 3b). Miniasm and raven did not significantly differ in ${E}_{INDEL}$. For ${E}_{combined}$, again a significant difference was observed between the four assemblers with canu performing worse compared to the other three assemblers, and once more, flye performing better than miniasm (Z = −2.64 and *p* < 0.05). ${E}_{combined}$ of miniasm and raven was not statistically significant.

#### No single polisher clearly outperformed the others

To decide whether one polisher statistically outperformed others, ${E}_{SNP}$, ${E}_{INDEL}$, and ${E}_{combined}$ of the different polisher were investigated (Fig. 3c, d). As flye performed best, in the following polishing of flye assemblies was evaluated. In general, we observed that polished assemblies of the *comparison group* showed significantly fewer errors than the *selection group* (${E}_{SNP}:Z= -2.26$, ${E}_{INDEL}: Z= -3.51$, and ${E}_{combined}: \text{Z }= - 3.04$, *p* < 0.05).

There was no statistically significant difference globally across all polishers for ${E}_{SNP}$, ${E}_{INDEL}$, and ${E}_{combined}$ considering all strains. Still, pairwise differences could be identified: Racon performed significantly worse than the other polishers considering ${E}_{INDEL}$ (clair3 Z =—2.78, medaka Z =—2.82, medakaBM Z =—3.44, and nextPolishLR Z =—2.74 with *p* < 0.05). There were no significant differences between any of the other polishers observed. Considering ${E}_{combined}$, only one local difference was observed. Racon performed worse compared to medakaBM (Z =—2.69, *p* < 0.05).

#### Long-read polishing of the assembly does not always improve the assembly accuracy

**Fig. 4:**
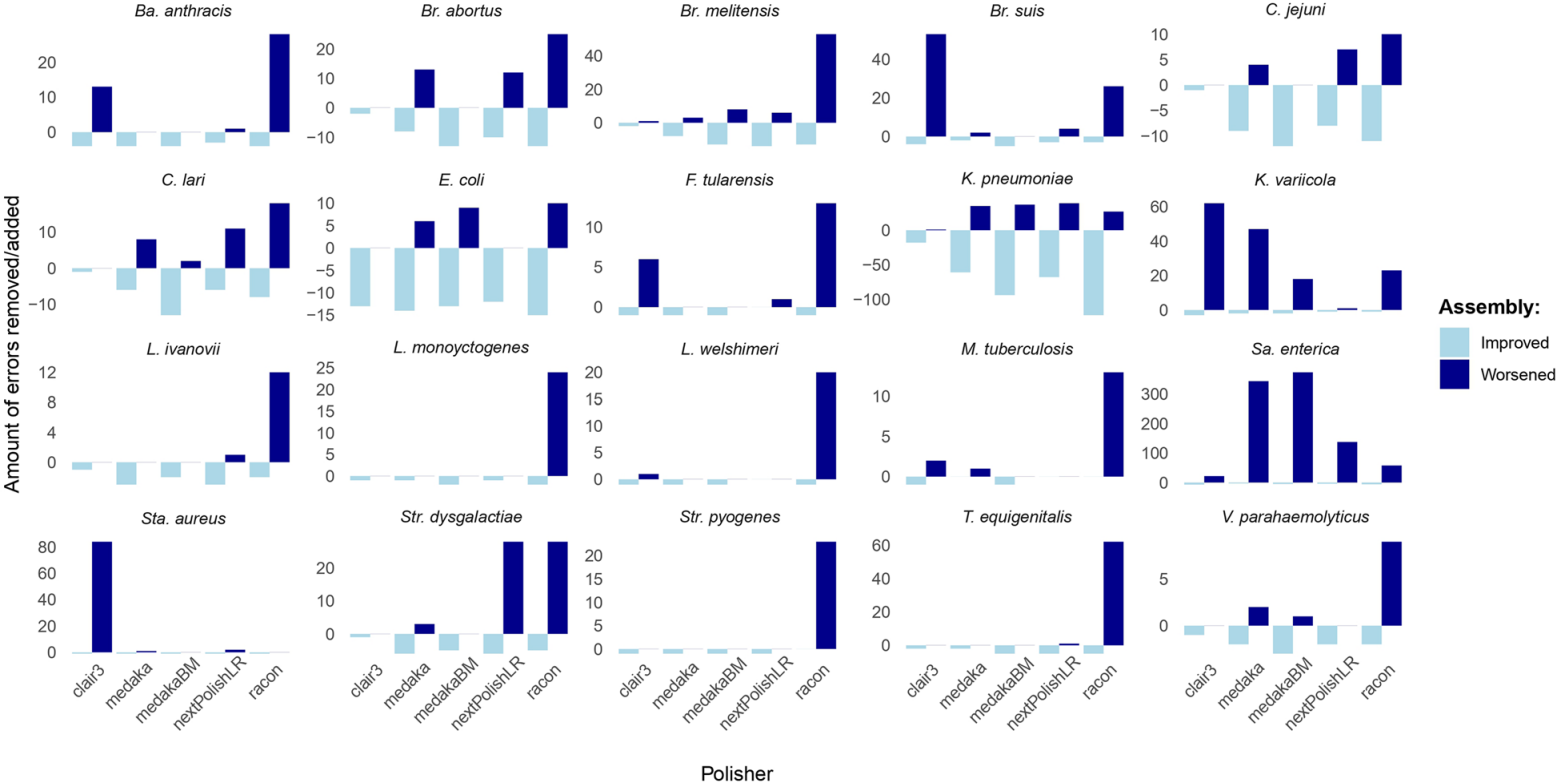
Impact of the different polishers on the accuracy of the flye assembly and one round of long-read polishing. Bar plots for each strain showing the influence of polishing on the assembly; improving the accuracy by correcting errors while polishing (light blue) or decreasing the accuracy by adding an error during polishing (dark blue)

Next, we analyzed whether polishing could also decrease the accuracy of the assembly. Here, the accuracy of the raw assemblies was compared to the accuracy of the polished assemblies. As in most cases, one polishing round was enough to reach the minimum ${E}_{combined}$ (Tables 2 and S2), we considered only one round of polishing. If a new error was introduced in the polished version, this was counted as worsening. If an error was detected within the unpolished assembly but not in the polished version, this was counted as improvement. If a homopolymer was corrected in length during polishing, but not completely correct, this was still considered as an improvement as the number of wrong bases was decreased. The analysis was conducted for flye assemblies and all possible polishers (Fig. 4). The results indicated that polishing with long reads can reduce the assembly accuracy across all polishers and species. In particular, racon was performing worse when considering insertions and deletions (INDELs), as already stated in the section before. Furthermore, the assembly of *Sa. enterica* was worsened substantially by medakaBM. MedakaBM introduced 374 additional errors into the assembly while correcting only four.

#### Errors were located mainly within coding sequences

**Fig. 5:**
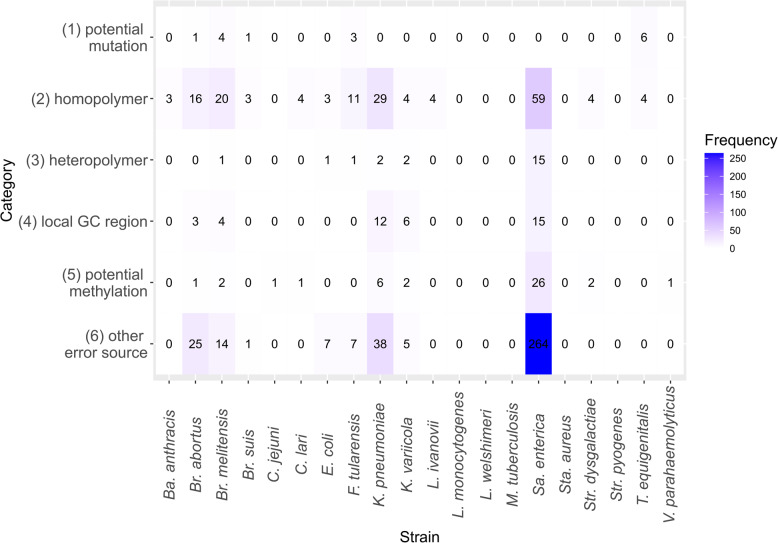
Heatmap of the different categories of differences ${E}_{combined}$ per strain. (1) Potential mutations, (2) homopolymers, (3) heteropolymers, (4) local high GC content region, (5) potential methylation-induced errors, (6) other errors

**Fig. 6:**
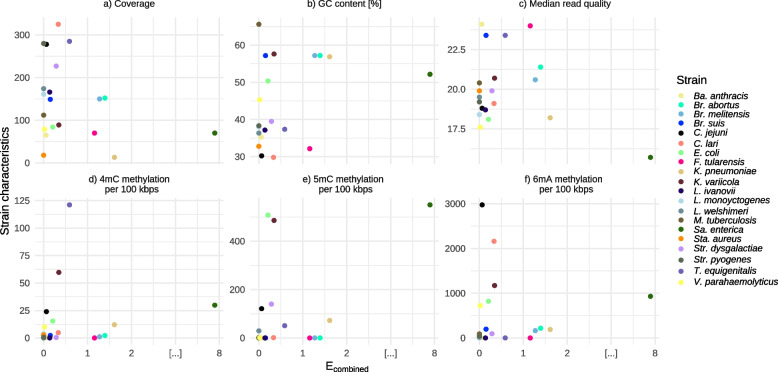
Influence of strain characteristics on ${E}_{combined}$. Sequencing and genomic characteristics (Y-axis) and ${E}_{combined}$ (X-axis) of the different strains

As flye did result overall in significantly fewer errors overall, and medaka with the bacterial methylation model (medakaBM) was most often part of the optimal strategy, this was chosen as a fitting default strategy for bacterial long-read genome assembly (supplement files 2: Default). To examine the potential error pattern of the default strategy, the type of errors and their genomic positions were further examined. To investigate whether strains showed specific error patterns, ONT and Illumina reads were mapped to the reference to investigate the errors at read level. We divided the differences between the assembly and reference into six distinct categories (Fig. 5): (1) potential biological mutations, where ONT and Illumina reads both covered for 95% the alternative base (differing to the base within the reference genome) and the coverage > 30X (2) homopolymer, a consecutive repetition of k times the same base with k > 2, (3) heteropolymer, a consecutive repetition of k times the same couple of bases XY with X! = Y and k > = 2, (4) a local high GC content region, where the proportion of “G” and “C” nucleotides was > = 0.7 within a 10 bp window around the error, (5) potential methylation-induced errors, where either the error was within an identified methylation motif or within ± five bp of a potentially methylated base, and (6) other errors, which included all errors that did not fall into any of the specified categories. Furthermore, the influence of sequencing and genomic characteristics was investigated (Fig. 6).

Five strains* (L. monocytogenes, L. welshimeri, M. tuberculosis, Sta. aureus,* and *Str. pyogenes)* resulted in the perfect genome reconstruction for flye with medakaBM (Table S2). Across all other strains, 80.8% of the differences were located within coding sequences (CDS). The average coding density across all strains were 89.3%. We further observed several potential mutations (15 differences; 2.3% of all differences) after recultivation of the strains, especially for *Br. melitensis* and *T. equigenitalis.* Additionally, 164 differences (25.6%) were attributed to homopolymers, e.g. for *Ba. anthracis*, the errors included two deletions of"A"s within homopolymers of length 19 and 38 bp. For *Br. melitensis*, both *Klebsiella* species*, Sa. enterica, F. tularensis*, and *E. coli* 22 differences (3.4% of all differences), respectively, were located within heteropolymers of length four to eight. Overall, 40 differences (6.2% of all differences) were located within local high GC content regions, especially for the two *Klebsiella* species (6 for *K. variicola,* and 12 for *K. penumoniae*) and *Sa. enterica* (15).

Figure 5b shows that median read quality of *Sa. enterica* was the lowest observed in the data set and led to the lowest ${E}_{combined}$ for the default strategy. Additionally, a greater fraction of methylated bases was observed within *Sa. enterica. Sa. enterica* has the highest level of detected 5mC methylation based on dorado. When checking for correlation, the analysis revealed a moderate positive correlation (*r* = 0.53) between the percentage of 5mC and combined error rates, indicating a tendency for errors to increase with higher levels of 5mC. This correlation was statistically significant ($p<0.05$), suggesting a meaningful relationship between these variables, with a confidence interval (*CI* = [0.12, 0.79]). For *K. pneumoniae* the coverage was 15 and less for all of the incorrect positions. All other strains did show sufficient coverage at positions categorized as potential assembly errors (> 30x). We checked for correlation and found a weak negative correlation (*r* = −0.25) between estimated coverage and ${E}_{combined}$, indicating a slight tendency for errors to decrease as coverage increases. However, the correlation was not statistically significant ($p>0.05$), with a wide confidence interval (*CI* = [−0.63, 0.21]). In particular, no clear connection between high GC content and low ${E}_{combined}$ could be observed. Nevertheless, all strains with rather low GC content resulted in lower ${E}_{combined}$. The test for correlation revealed a weak positive correlation (*r* = 0.26) between GC content and ${E}_{combined}$. However, this relationship was not statistically significant ($p>0.05$), and the confidence interval (*CI* = [−0.21, 0.63]) again indicated uncertainty of the observed correlation.

#### Methylation motifs and methylated bases can result in a portion of the reads having ambiguous bases in or near methylated regions

In nanopore sequencing, basecalling is influenced by neighbouring bases, as the signal is generated by a k-mer of nucleotides passing through the nanopore rather than individual bases. This can result in bases near a methylated base being incorrectly basecalled [34]. Now, if the proportion of reads with the wrong basecall outweighs the proportion of reads with the correct basecall, the error may end up in the final de novo assembly, which is essentially a consensus sequence based on the sequenced reads. We detected methylation using dorado basecalling with methylation-specific models, followed by analysis with Modkit. Then, we compared the positions of errors in the genome assemblies to those of detected methylated motifs and methylated sites (nucleotides). For methylated sites, we allowed a window of ± five base pairs based on previous studies that showed that the methylation signal might not directly impact the same base being mis-basecalled, but rather affect multiple bases in the surrounding area [28, 29]. Across all strains, 42 (6.5%) of the differences were potentially caused by methylation. We found different methylated motifs; not all of these motifs were associated with assembly errors. When assembly errors were linked to a detected motif, only specific occurrences of the motif were affected and the motif did not always lead to erroneous basecalls.

For example, in *V. parahaemolyticus*, the motif CTACNNNNNNNTCG contained a methylated adenine (6mA) on the reverse strand (corresponding to a “T” on the positive strand at position 12 of the motif). In one motif occurrence in the genome assembly, this methylated base caused the incorrect basecalling of a “G” on the reverse strand, two positions upstream (position 14 in the motif), as a cytosine. In *K. pneumoniae*, six errors were located within the GATC motif, while in *Sa. enterica* also two errors were similarly associated with the same motif. The motif CGACNNNNNGGT, found in *Br. melitensis*, has two methylated adenines: one on the third position on the positive strand and one at the last position on the reverse strand. Both methylated positions were within five bp of an incorrect basecall (“G” (in the reference sequence) being included as “T” (in the assembly). We found the motif GANTC in *Br. melitensis* and *Br. abortus*, associated with two potential methylation-related errors.

In other cases, errors occurred near methylated bases without the presence of a specific detected motif. For example, in *Str. dysgalactiae*, a potentially methylated “C” (5mC) three bases downstream of a reference cytosine coincided with basecalling variability at this position (14 reads called as “A”, 298 reads as “C”, 141 reads as “T”). In *K. variicola*, a methylated “C” (5mC) two bases downstream of a “T” (reference) resulted in incorrect basecalling (32 reads as “C”, 91 reads as “T”) and an incorrect “C” in the assembly at this position. Similarly, in *C. lari,* a methylated “A” (6mA) two bases upstream of a reference “C” resulted in a “T” in the assembly, with reads covering “C” 876 times and “T” 374 times.

When comparing the errors left in the assembly with the v500 medaka model compared to the medaka methylation-aware model (both used the same reads, basecalled with model dna_r10.4.1_e8.2_400bps_sup@v5.0.0, and the same assembly), a reduction of errors could be observed. The medaka v500 model resulted in 90 errors within the assemblies, all linked to a methylated base. In contrast, the methylation-aware model only produced 42 errors linked to a methylated base.

### How do the detected errors impact high-resolution genotyping (cgMLST)?

**Fig. 7:**
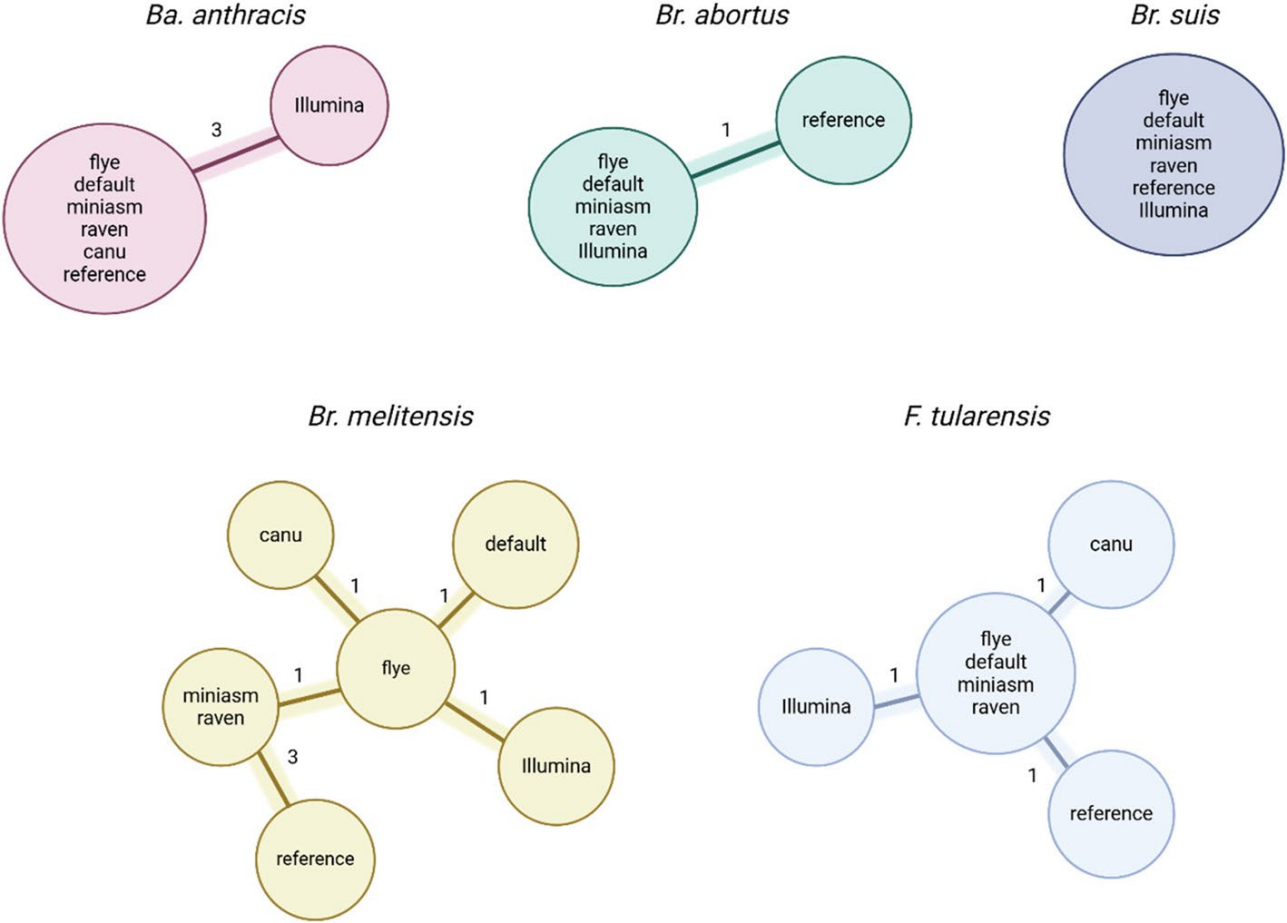
Results for cgMLST. Minimum spanning trees for *Ba. anthracis*, *Br. abortus*, *Br. suis*, *Br. melitensis,* and *F. tularensis*. Reference, Illumina assembly, best possible assembly for every assembler, and the default strategy (best overall strategy: flye + medakaBM) were used. The canu assembly was excluded for *Br. abortus* as more than 10% of the loci were missing. The assembly process of canu for *Br. suis* failed. CgMLST analysis was performed with SeqSphere + and clusters were defined with a maximum of five allelic differences and are highlighted in color, with each branch indicating the number of allele differences

Next, we investigated whether these errors in the assemblies do influence the accuracy of high-resolution genotyping, in particular cgMLST. Hence, for each assembler the most accurate assembly plus the defined default strategy (flye + medakaBM) were analysed together with an Illumina-only assembly and the reference genome (Fig. 7). The *Ba. anthracis* cgMLST comprises 3,803 predefined cgMLST targets [19], 1,764 cgMLST targets are included in the scheme for *Brucella* spp. [35], and the *F. tularensis* cgMLST scheme contains 1,147 predefined cgMLST targets [18]. Clusters were defined with a maximum of five allelic differences. As all assemblies were created from the same DNA, in an accurate scenario all assemblies should cluster together with no difference.

#### While some strains resulted in perfect clustering, others included allele differences despite resulting from the same sequencing reads

For *Ba. anthracis* all analysed long-read assemblies did not differ in the cgMLST loci compared to the reference, only the Illumina assembly differed in three loci. Furthermore, for *Br. abortus,* all assemblies (long-read and Illumina) differed in the same loci compared to the reference. For *Br. suis*, all assemblies were identical in the cgMLST loci. A different scenario emerged for the third *Brucella* species. For *Br. melitensis*, the raven and miniasm assemblies differed in three loci compared to the reference genome, the flye assembly in one additional locus. The default strategy assembly, the Illumina assembly, and the canu assembly differed in total in five loci compared to the reference, which would still be considered the same outbreak cluster as the threshold was set to five allele differences. For *F. tularensis*, the Illumina assembly, the reference, and the canu assembly differed in one locus compared to the other long-read assemblies.

The observed allele difference between the analyzed *Br. abortus* strain and its corresponding reference sequence is likely caused by a mutation, as all assemblies (short and long-read) did include the same allele difference compared to the reference genome. For *Ba. anthracis*, the three Illumina loci differing from the rest did have insufficient coverages of 2–8X in the corresponding position leading to incorrect assembling. The same applies for *F. tularensis*. Here, the position was sufficiently covered by ONT reads (67X), but only by three Illumina reads leading to a “T” for Illumina, instead of a “G” (reference and ONT assemblies). For the SNP between Illumina and the reference compared to the ONT assemblies in *Br. melitensis*, ONT reads covered both bases (reference and Illumina assembly: “C” and ONT assemblies: “G”) with equal reads leading to a “G”, while Illumina more reads (44 x “C” to 31 x “G”) confirmed the reference base “C”.

## Discussion

Oxford Nanopore Technologies (ONT) sequencing is increasingly utilized in the field of microbial genomics because of its capability to perform long-read and real-time sequencing [13]. However, studies have shown that the success of nanopore sequencing varies significantly across different bacterial species, especially for outbreak analysis. This study aims to address these discrepancies by evaluating the performance and applicability of ONT sequencing for genome reconstruction across various bacterial species, with the goal of investigating its foundation for pathogen surveillance and determining its potential for broader use in monitoring and controlling infectious disease outbreaks.

ONT is continually improving its chemicals and devices [36], as well as bioinformatics software, enhancing accuracy and usability, but not all bacterial species benefit equally from these advancements. Among the evaluated basecallers, dorado duplex emerged as the most accurate but also requires the largest computational resources and computation time (duplex used on average 72.4% more resident set size (RSS) and 25.6% more CPU time for our sequencing data), potentially limiting its practical usage. It is worth noting that ONT released Dorado v0.9.6 on April 16, 2025, which is reported to offer improved computational efficiency and faster basecalling. As a result, the performance trade-offs observed in our analysis may no longer hold with the latest version. In certain rare instances, dorado simplex basecalling called fewer incorrect positions compared to dorado duplex basecalling (1 to 13 positions). The affected positions were characterized by multiple reads supporting incorrect bases. Notably, the simplex dataset presented a greater relative proportion of reads correctly supporting the reference base, whereas the duplex dataset, despite slightly greater coverage, exhibited a greater proportion of reads indicating an alternative base. E.g. for *Br. abortus* (alternative base “T” instead of reference base “C” for duplex), for dorado simplex 99 reads supported “C” and 76 reads “T”, whereas for dorado duplex 104 reads supported “C” and 83 reads “T”. The different basecalling models did show an improvement overall, but not across all species. For instance, *Br. abortus* has not benefited in the same level of model optimization throughout the models. Basecalling heavily depends on neural networks, and if a species is not included in the training set, it can lead to significant errors. This is especially pertinent for species classified as Biosafety Level 3 (such as all strains in the *selection group* except *T. equigenitalis*, and *M. tuberculosis* from the *comparison group*), which are unlikely to be represented in training datasets due to handling restrictions. In contrast, more common pathogens such as *Campylobacter* spp. or *Listeria* spp. are likely to be included in these datasets because of their public health relevance, resulting in more reliable basecalling performance for these species. Our hypothesis is supported by the result that assemblies from the *selection group* demonstrated a significantly higher error rate compared to those from the *comparison group*. This could also be because the hybrid assemblies used as references might contain sequencing errors. More detailed information regarding which bacterial species were used for model-training, would advance transparency and improve the interpretation of species-specific error patterns.

In general, flye produced the most accurate, continuous, and complete assemblies, which is consistent with findings from other studies [37, 38], whereas others recommended alternatives such as raven [39]. Although, some inversions have occurred, assemblies additionally often lack a standardized starting point. Hence, it is advisable to reorient contigs after assembly for downstream analysis [40–42]. Flye was unable to assemble two small plasmids for *E. coli* and instead created a new 7200 bp long fragment, and the assembly did include inversions. The tendency of flye not to assemble small plasmids was previously noted by Johnson et al. [43]. Canu, on the other hand, produced incomplete, duplicated assemblies that could lead to inaccurate epidemiological interpretations. This could be caused by canu’s design to reconstruct mammalian genomes [44]. Regarding polishing, the latest medaka model, specifically designed to be bacterial methylation-aware, showed the most promising results, although it does not always result in the most accurate assembly. Previous research indicates that species-specific or closely related species-trained models lead to better results, reflecting the similar DNA modifications in these organisms [31]. This underscores the superior performance of bacterial-specific models such as r1041_e82_400bps_bacterial_methylation from medaka, which also relies on neural networks. Interestingly, while multiple assembly-polisher strategies yielded the best overall results, performing polishing occasionally worsened assembly quality. However, the lack of conclusive results suggests that there is no universal solution, as different assemblers and polishers affect results variably across species sequencing characteristics. Considering the limited sample size of 20 strains, the statistical tests have reduced power, potentially affecting their universal applicability. The small sample size can limit the ability to detect significant error patterns and may lead to by chance incorrect conclusions. In addition, other studies have reported these differences in performance [37, 45], with Wick et al. suggesting a consensus approach using different assemblies combined with Trycycler [46], which could be a viable option for smaller datasets, where manual curation is feasible.

Mutations arising during recultivation introduced discrepancies between the sequenced isolate and the reference genome for the *selection group*. This issue is a biological process and no technical problem, as Illumina data supported these positions as well and therefore ruling out ONT specific sequencing error. On the other hand, errors in homopolymeric regions remain a known challenge for ONT sequencing, as the electrical signal generated while DNA passes through the nanopore does not vary significantly with repeated identical bases [7]. This intrinsic limitation was evident in our study and accounted for 25% of the errors. An earlier study from 2021 (with R9.4.1 flowcells) found that nearly half of the errors were attributed to homopolymers [7], indicating improvements in ONT sequencing (from R9.4.1 to R10.4.1 flowcells) but highlighting it as a persistent systematic error. A small proportion of errors (3.4%) were located within heteropolymers, indicating that these are not the main error source. This is in particular interesting as these are often part of regulatory regions [47] or used for genotyping based on multilocus variable number of tandem repeat analysis (MLVA) [48]. Local high GC content regions caused 6% of the errors, indicating that there is no strong correlation between global high GC content and an increased error rate, still local regions could be a cause for assembly errors. GC content as a potential error source was also reported by Delahaye [7].

A special case was *Sa. enterica*. The unpolished flye assembly initially contained nine errors, six of which were located in high-GC content regions, whereas three occurred in homopolymeric regions. Following long-read polishing with medakaBM, the number of errors increased to 379. Among these, five errors persisted from the unpolished version, four in high-GC content regions and one in a homopolymeric region. However, the significant majority of the errors introduced during polishing were new, highlighting a limitation in the medakaBM model. One reason might be data quality as reads were short and had rather low sequencing quality (median 15.7). Both of these factors, together with the high proportion of methylated bases, could lead to the high number of errors.

One source of incorrect basecalling and consequently incorrect genome assembly was methylation, which has been identified to lead to ambiguous bases. Such a correlation between increased methylation and increased error rate has been shown in recent studies [26–30]. Due to the ambiguous bases caused by methylation polishers introduce errors, an issue that the medaka model apparently did not exacerbate to certain extend. Lohde et al. [28] demonstrated that some strains pose significant challenges for methylation, while others perform exceptionally well [30]. In this study, a relatively low error rate due to methylation (6.5%) was observed. This might be due to the new polishing model (medakaBM) which was not available during the analysis of Dabernig-Heinz et al. [20]. Moreover, authors stated that the number methylation based strongly varies between different strains of one species [28], whereas only one strain per species was used in this study. This restricts the ability to capture intraspecies variation, a factor that could influence accuracy, as highlighted by Dabernig-Heinz et al. [27]. Especially, these methylation patterns remain challenging as gold standard methods to identify methylations are not present.

The identified methylation motifs GATC [49] and GANTC [50] are well-known methylation patterns. The corresponding DNA-methyltransferase Ccrm was found in the assemblies of *Br. abortus* and *Br. melitensis*. Nevertheless, methylated motifs and methylated bases did not lead to errors in most cases, indicating that current basecalling models can already handle this source of errors relatively well. Compared to the medaka model v500, the methylation-aware model resulted in 48 errors fewer linked to methylation, indicating an improvement in accuracy.

Our findings revealed that 81% of the observed errors occurred within CDS (with an average coding density of 89.3% across all strains), potentially compromising the accuracy and reliability of the cgMLST results. While the focus of this study was on optimal assembly strategies and detailed error analysis, in a previous study [23], we showed that cgMLST based ONT data are comparable to Illumina data for *F. tularensis* and *Ba. anthracis,* but not for *Br. suis*. However, this previous study [23] used outdated flow-cells and bioinformatics analysis. In this study, all ONT based assemblies presented fewer than five allele differences compared to the respective references. While a fixed threshold is an ongoing matter of debate, many studies used five alleles to define outbreak clusters [19, 23, 51]. In particular, cgMLST loci differ due to mutation, insufficient Illumina coverage, and ambiguous read coverage.

Low sequencing depth caused errors for *K. pneumoniae*, which is consistent with findings from other studies suggesting a minimum coverage threshold of 30X—50X [7, 20]. However, as seen with simplex outperforming duplex basecalling in some rare cases, higher coverage does not always imply more accurate assemblies, especially with ambiguous bases. Furthermore, some of these errors remain unaffected by greater sequencing depth due to their systemic nature. Methylation-related errors, for example, cannot be corrected by increasing the depth of sequencing. Lohde et al. [28] suggested masking regions with ambiguous sites, although this approach might lead to the loss of valuable information. An alternative solution could be PCR-based library preparation, although this would come at the cost of potentially shortening the read length and losing data on methylation and other modifications.

## Conclusion

In this study, six bacterial strains with low mutation rates were sequenced using Oxford Nanopore Technologies (ONT) alongside their Sanger-sequenced reference genomes to evaluate various methods for assembly reconstruction. The dataset was further expanded by incorporating publicly available data, including 14 pathogens relevant to public health and the food sector. Tool performance varied notably across assemblers, polishers, and species. While some species achieved perfect genome assemblies, others presented numerous inaccuracies in nucleotide positions. The assemblies exhibited errors related to homopolymers, heteropolymers, local high GC content, low coverage or methylation, with identifiable methylation motifs. Here, the bacterial methylation-aware model of medaka improved the accuracy of the assemblies. Our data indicated, that one round of polishing is sufficient in most cases, and in rare cases that polishing might worsen the assembly quality. Further advancements in basecalling accuracy could improve performance, particularly for bacteria that are less extensively studied or classified as biosafety level 3 organisms. 81% of the errors occurred in CDS, and thus, potentially within cgMLST loci. While the number of errors remained below typically applied thresholds in this study, a single isolate may not always be consistently assigned to the same outbreak cluster.

## Materials and methods

### Strain selection

#### Selection group with RefSeq reference genomes

Strains were selected based on their pathogenicity, low mutation rates, and the availability of a Sanger-sequenced reference genomes (Table 4). The reference genomes were downloaded from the NCBI RefSeq database. The following reference strains were selected: *Bacillus anthracis* Ames Ancestor, *Brucella abortus* bv. 2 86/8/59, *Brucella melitensis* 16M, *Brucella suis* ATCC 23445, *Francisella tularensis* LVS, and *Taylorella equigenitalis* ATCC 35865.

**Table 4:** Strain selection with run accession numbers for ONT reads, Illumina reads, and the reference genome

Strain	Run accession number ONT	Run accession number Illumina	Reference genome
***Selection group***			
*Bacillus (Ba.) anthracis* Ames Ancestor	ERR14031228	ERR14879208	GCF_000008445.1
*Brucella (Br.) abortus* bv. 2 86/8/59	ERR14031225	ERR14879205	GCF_000740375.1
*Brucella (Br.) melitensis* 16M	ERR14031226	ERR14879206	GCF_000740415.1
*Brucella (Br.) suis* ATCC 23445	ERR14031227	ERR14879207	GCF_000018905.1
*Francisella (F.)* tularensis LVS	ERR14031230	ERR14879210	GCF_000833335.1
*Taylorella (T.) equigenitalis* ATCC 35865	ERR14031229	ERR14879209	GCF_900637125.1
***Comparison group***			
*Campylobacter (C.) jejuni* ATCC-33560	SRR28370641	SRR26899120	GCF_045689445.1
*Campylobacter (C.) lari* ATCC-35221	SRR28370640	SRR26899115	GCF_045689455.1
*Escherichia (E.) coli* ATCC-25922	SRR28370642	SRR26899128	GCF_045288035.1
*Klebsiella (K.) pneumoniae* KPC2	SRR28370681	SRR28370701	GCF_045345375.1
*Klebsiella (K.) variicola* AJ292	SRR28370683	SRR28370702	GCF_045345325.1
*Listeria (L.) ivanovii* ATCC-19119	SRR28370639	SRR26899136	GCF_045288005.1
*Listeria (L.) monocytogenes* ATCC-BAA-679	SRR28370636	SRR26899101	GCF_045288145.1
*Listeria (L.) welshimeri* ATCC-35897	SRR28370637	SRR26899109	GCF_045689465.1
*Mycobacterium (M.) tuberculosis* mc26030	SRR28370678	SRR28370698	GCF_045345655.1
*Salmonella (Sa.) enterica* ATCC-10708	SRR28370644	SRR26899135	GCF_045287905.1
*Staphylococcus (Sta.) aureus* BPH2947	SRR28370684	ERR2929425	GCF_045345275.1
*Streptococcus (Str.) dysgalactiae* STG643	SRR28370679	SRR28370699	GCF_045345565.1
*Streptococcus (Str.) pyogenes* BPH2947	SRR28370680	SRR28370700	GCF_045345455.1
*Vibrio (V.) parahaemolyticus* ATCC-17802	SRR28370643	SRR26899141	GCF_045287955.1

#### *Comparison group* (Hall et al. dataset)

Additionally, a publicly available sequencing dataset (ONT, Illumina, and manually curated hybrid assemblies) of 14 common pathogenic bacteria (Table 4) was downloaded (BioProject PRJNA1087001, Hall et al. [32]). This dataset was included to test the approaches for a broader spectrum of bacteria, especially important within health and food safety sector.

### Cultivation and sequencing

#### Cultivation and DNA extraction

*Ba. anthracis* strain was cultivated on nutrient agar (Merck, Darmstadt, Germany) for 24 h at 37 °C and DNA was extracted using the Genomic-tip 100/G kit (Qiagen, Hilden, Germany). *Brucella* sp. (*Br. melitensis*, *Br. abortus* and *Br. suis* biovar 2) were grown on nutrient agar with 7.5% calf blood for 48 h at 37 °C. For *T. equigenitalis* cultivation, modified Eugon chocolate agar supplemented with 5 mg/L amphotericin B (MECA + A) was used and the plates were incubated at 37 °C with 5% CO_2_ addition for 72 h. DNA was isolated from inactivated biomass of the four species with the NucleoBond HMW DNA kit (MACHEREY–NAGEL, Düren, Germany) for high-molecular DNA. *F. tularensis* was cultivated on cysteine heart agar (CHA, Becton Dickinson, BD Heidelberg, Germany) at 37 °C with 5% CO2 for 72 h. The DNA used for whole genome sequencing was prepared using the QIAGEN Genomic-tip 20/G Kit (Qiagen GmbH, Hilden, Germany). The DNA extraction was performed according to the instructions of the manufacturer for sample preparation and the lysis protocol for bacteria using 1 ml buffer B1 with 2 μl RNase A, 45 μl proteinase K, and 20 μl lysozyme.

#### Sequencing

All six strains were sequenced with a MinION Mk1B device from Oxford Nanopore Technologies (Oxford, United Kingdom) and, additionally, Illumina MiSeq® (Illumina Inc., San Diego, CA, USA). Nanopore sequencing library preparation and barcoding were performed with the Native Barcoding Kit 24 V14 (SQK-NBD114.24). The sequencing was performed with a R10.4.1 flow cell (FLO-MIN114) according to the manufacturer’s instructions.

For Illumina sequencing, the DNA of the six strains was extracted and purified using the QIAGEN® Genomic-tip 20/G Kit (QIAGEN, Germany) and the Genomic DNA Buffer Set (QIAGEN, Germany). The concentration of the DNA was determined using the Qubit dsDNA BR assay kit (Invitrogen, United States). Sequencing libraries were created using the Nextera XT DNA Library Preparation Kit (Illumina Inc., United States). Paired-end sequencing with two cycles of 301 bps was performed on an Illumina MiSeq instrument according to the manufacturer's instructions (Illumina Inc., United States).

### Bioinformatic analysis

#### Genome reconstruction

The different steps and tools were selected based on their common use and availability on conda (Table 2). For the analysis, a snakemake workflow was applied (https://gitlab.com/FLI_Bioinfo/BONT, version 1.0.0). All analyses were performed on a cluster equipped with Intel Xeon Gold 6240 CPUs (380 GB memory) and 2 GPU (NVIDIA Tesla V100S-PCIE-32GB) together with SLURM for job scheduling. All tool parameters are stated in supplement file 2: settings. Dorado v0.8.2 (with models dna_r10.4.1_e8.2_400bps_sup@v4.2.0, dna_r10.4.1_e8.2_400bps_sup@v4.3.0, and dna_r10.4.1_e8.2_400bps_sup@v5.0.0) [52] and guppy v6.5.7 (with model dna_r10.4.1_e8.2_400bps_sup.cfg) [53] were applied for basecalling, and filtlong v0.2.1 [54] for filtering. NanoPlot v1.42.0 [55] and Quast v5.2.0 [56] for quality assessment. Assemblers canu v2.2 [44], flye v2.9.3 [38], miniasm v0.3 [57], and raven v1.8.3 [58] were used. For polishing, clair3 v1.0.5 (with the respective models to the basecalling models) [59], medaka v1.12.0 (with the respective models to the basecalling models, and additionally the bacterial methylation model r1041_e82_400bps_bacterial_methylation) [60], nextPolish v1.4.1 [61], and racon v1.4.3 [62] were evaluated. BUSCO v5.7.1 [63] was used to investigate completeness of the final assemblies, with reference set indicated in supplement file 1, BUSCO. For statistical analysis, the Shapiro–Wilk test was used to check for normal distribution, the rates ${E}_{SNP}$, ${E}_{INDEL}$, and ${E}_{combined}$ did not follow normal distribution ($W=0.32, p < 2.2{e}^{-16}$, $W=0.32, p < 2.2{e}^{-16}$; and $W=0.32, p < 2.2{e}^{-16}$, respectively). The Kruskal–Wallis test was applied to investigate significant differences globally across different groups, while the Dunn’s test with Bonferroni correction was applied for pairwise comparison (here, Z stands for many standard deviations the observed value is away from the expected value under the null hypothesis; which assumes no differences between the two groups with $p<0.05$). The correlation was determined using Pearson's product-moment correlation coefficient.

#### Evaluation of errors

Criteria for evaluation were various kinds of differences between the final assembly and the reference genome. Thus, SNPs and INDELs were identified with Quast v5.2.0. ONT and Illumina reads were mapped to the reference genome with minimap2 v2.26 [64], and samtools v1.19.2 [65] to access coverage and nucleotide abundance on read level. Annotation of the reference genomes was performed with bakta v1.7.0 [66]. Analysis was performed with R, plots were created with ggplot2 [67].

In this context, the terms *SNP* (base substitution), *Ins* (insertion) and *Del* (deletion) refer to instances where the nucleotide in the final assembly differs from the nucleotide at the corresponding position in the reference genome.

The *combined error rate *${E}_{combined}$ is then defined as the amount of differences compared to the reference, normalized to the rate per 100 kbps due to different genome sizes:

1 $${E}_{combined}\left(AS(A,P,r,i)\right)= \frac{{SNP}_{AS(A,P,r,i)}+In{s}_{AS(A,P,r,i)}+ De{l}_{AS(A,P,r,i)}}{{G}_{i}}*100\ 000$$

where $AS(A,P,r,i)$ is the assembly *AS* built with assembler $A$ for strain $i, i\in S, S$ including the strains of the two different groups and polished with polisher $P$ at round $r$. ${SNP}_{AS(A,P,r,i)}$ represent the number of SNPs for assembly $AS(A,P,r,i)$, ${Ins}_{AS(A,P,r,i)}$ represent the number of insertions for assembly $AS(A,P,r,i)$, ${Del}_{AS(A,P,r,i)}$ represent the number of deletions for assembly $AS(A,P,r,i)$*,* and ${G}_{i}$ the genome size of corresponding strain $i$.

The assembly approaches were evaluated with the combined error rate. The strategy was determined on the basis of:

2 $$AS^\ast\left(A,P,r,i\right)=arg\;\underset{}{min_P\mathit\;}E_{combined}\left(AS\left(A,P,r,i\right)\right)$$

where $A{S}^{*}(A,P,r,i)$ is the best assembly built with assembler $A\in \left\{canu, flye, miniasm,raven\right\}$ of strain $i$, after $r-1$ rounds of polishing (so $A{S}^{*}(A,P,r-1,i)$), polished in round $r$ by the optimal polisher $P$, with $P\in \left\{clair3, medaka, medakaBM, nextPolishLR, racon, noPolishing\right\}$.

Continuity was measured with the average deviation $\overline{Dev }(A{S}_{A})$ of the contigs compared to the reference:

3 $$Dev\left(A{S}_{A}\right)= C\left(A{S}_{A,i}\right)-C\left({R}_{i}\right)$$

4 $$\overline{Dev }(A{S}_{A})=\frac{{\sum }_{i \epsilon S}C(A{S}_{A,i})-C\left({R}_{i}\right)}{|S|}$$

where $S$ is the set of strains, $C\left(A{S}_{A,i}\right)$ is the number of contigs of the best possible assembly for strain *i* built with assembler $A$, and $C\left({R}_{i}\right)$ is the number of contigs of the reference for strain *i*.

To investigate whether the performance of assemblers and polishers differs, the rate of SNPs:

5 $${E}_{SNP}\left(AS\left(A,P,r,i\right)\right)= \frac{{SNP}_{AS\left(A,P,r,i\right)}}{{G}_{i}}*100\ 000$$

and the rate of insertions and deletions

6 $${E}_{INDEL}\left(AS\left(A,P,r,i\right)\right)= \frac{In{s}_{AS\left(A,P,r,i\right)}+ De{l}_{AS\left(A,P,r,i\right)}}{{G}_{i}}*100\ 000$$

and ${E}_{combined}$ (see (1)) were evaluated. Here, ${SNP}_{AS\left(A,P,r,i\right)}$, $In{s}_{AS\left(A,P,r,i\right)}$, and $De{l}_{AS\left(A,P,r,i\right)}$ are the number of SNPs, insertions and deletions, respectively, of the assembly built with assembler $A\in \left\{canu, flye, miniasm,raven\right\}$ of strain $i$, after $r-1$ rounds of polishing, polished in round $r$ by the polisher $P$, with $P\in \left\{clair3, medaka, medakaBM, nextPolishLR, racon, noPolishing\right\}$ compared to the reference. ${G}_{i}$ is the genome size of corresponding strain $i$.

#### Identification of methylation

Methylation was assessed with dorado v0.8.2 (basecalling with 6mA, 4mC_5mC models) and a Nextflow workflow to identify methylation motifs and methylated bases (available at https://github.com/valegale/ONT_methylation, version 0.0.1). In brief, the workflow used Modkit (https://github.com/nanoporetech/modkit) v0.4.1 to aggregate read information on methylation status and calculate the percent methylation for 6mA, 4mC, and 5mC. Positions with a high percentage of modification, greater than 0.5– calculated as the ratio of methylated reads to total coverage at a position– were classified as methylated. The workflow also included a recent feature of Modkit for reliably detecting motifs with high methylation frequency (95% to 100% in the whole genome). This feature can recognize short palindromic type II motifs and longer type I bipartite motifs.

#### cgMLST

Illumina reads were assembled using the pipeline WGSBAC (v 2.2.3) [68]. Here, the raw paired-end reads served as input, the coverage was calculated and the quality of the short-read sequencing data were assessed with FastQC [69]. A coverage > 30X was considered acceptable for further analyses. The quality and adapter trimming of the Illumina reads and assembly were performed by Shovill v. 1.0.4 [70].

The cgMLST analysis was performed with the software SeqSphere + [71] and the respective schemes for *Ba. anthracis* [19], *Brucella* spp. [35], and *F. tularensis* [18]. Graphs representing minimum spanning trees were created with BioRender. The cluster threshold was defined as five allelic differences for all species.

## Supplementary Information

Supplementary Material 1: Supplement Table S1. Quality measures of Illumina and ONT sequencing for all strains. Mean and median read length, mean and median read quality, percentage of reads > Q15, estimated coverage and GC content

Supplementary Material 2: Supplement Table S2. Extended results strategy evaluation. Table “Basecalling”, “Assembly”, and “Default” include amount of insertions, deletions, and substitutions, as well as $E_{combined}$ for basecalling, assembly, and polishers. Table “BUSCO” includes the BUSCO results for all assemblies. Table “Contigs” include the contig information for all assemblies. Table “Settings” includes all predefined settings of all tools used.

Supplementary Material 3: Figure S1. Evaluation of the basecaller and models influencing the accuracy the final assembly (*comparison group*). a) Violin plot of $E_{combined}$ of final assemblies for dorado duplex, dorado simplex, and guppy with model v420. b) Violin plot of $E_{combined}$ of final assemblies for dorado duplex using models v420, v430, and v500. Figure S2. Continuity of final assemblies for each assembler (all strains). The deviation $Dev\left(AS_A\right)$ (y-axis) for each strain and assembler. The white numbers indicate $Dev\left(AS_A\right)$, when $Dev\left(AS_A\right)>5$. An “X” indicates that the assembly process failed. The colors indicate the different assemblers (on the x-axis).

## Data Availability

The sequencing data used in this manuscript has been deposited with the European Nucleotide Archive and is available under Bioproject PRJEB83096 (ONT) (https://www.ebi.ac.uk/ena/browser/view/PRJEB83096) and Bioproject PRJEB88823 (Illumina) (https://www.ebi.ac.uk/ena/browser/view/PRJEB88823).
