# Supplementary material for: Accurately assembling nanopore sequencing data of highly pathogenic bacteria
Source: BMC Genomics. 2025 Aug 28;26:783. doi: 10.1186/s12864-025-11793-6 (PMC12392509; doi:10.1186/s12864-025-11793-6)
Supplement: Supplementary file 3 — Supplementary Material 3: Figure S1. Evaluation of the basecaller and models influencing the accuracy the final assembly (comparison group). a) Violin plot of $E_{combined}$ of final assemblies for dorado duplex, dorado simplex, and guppy with model v420. b) Violin plot of $E_{combined}$ of final assemblies for dorado duplex using models v420, v430, and v500. Figure S2. Continuity of final assemblies for each assembler (all strains). The deviation $Dev\left(AS_A\right)$ (y-axis) for each strain and assembler. The white numbers indicate $Dev\left(AS_A\right)$, when $Dev\left(AS_A\right)>5$. An “X” indicates that the assembly process failed. The colors indicate the different assemblers (on the x-axis). [file 12864_2025_11793_MOESM3_ESM.docx]

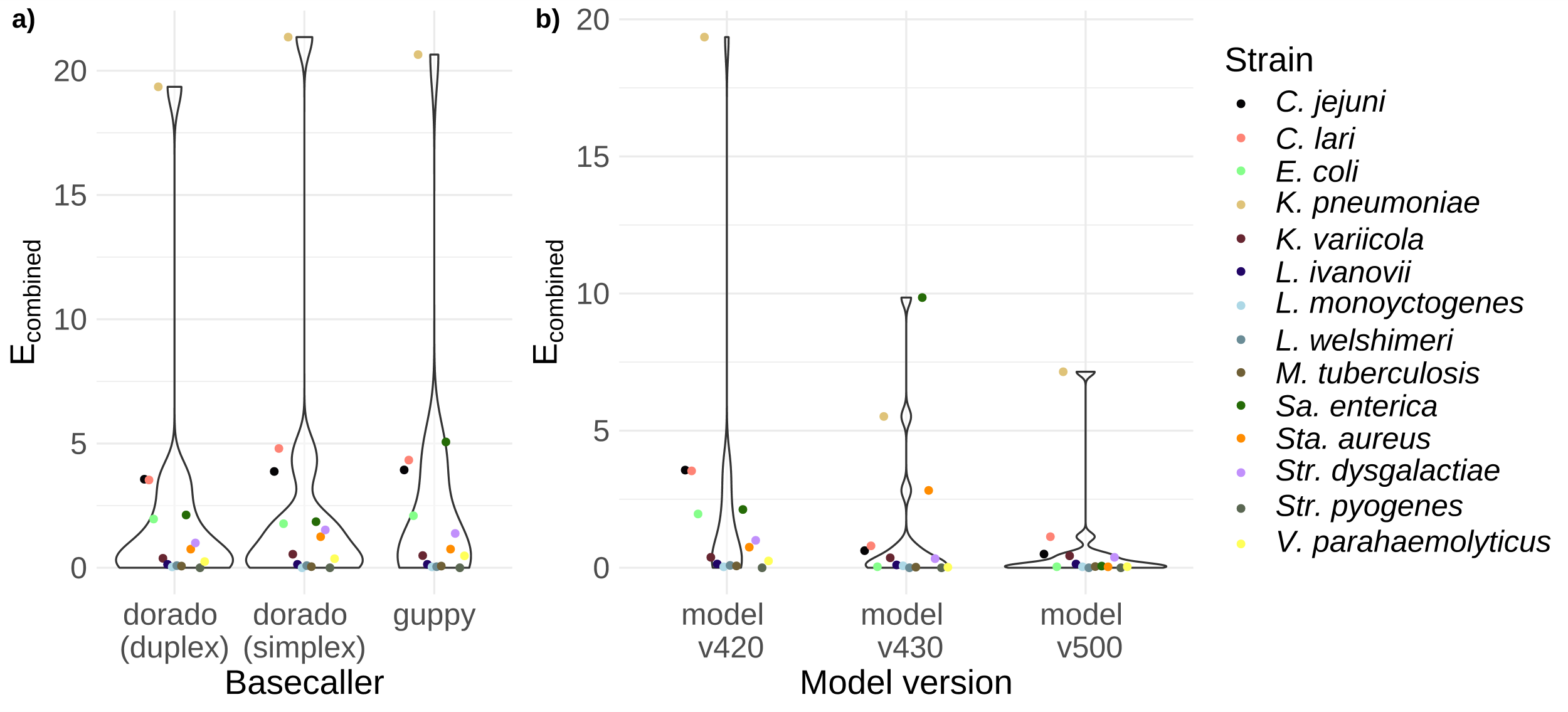


**Figure S1:** Evaluation of the basecaller and models influencing the accuracy the final assembly (*comparison group*). a) Violin plot of $E_{combinedError}$ of final assemblies for dorado duplex, dorado simplex, and guppy with model v420. b) Violin plot of $E_{combinedError}$ of final assemblies for dorado duplex using models v420, v430, and v500.


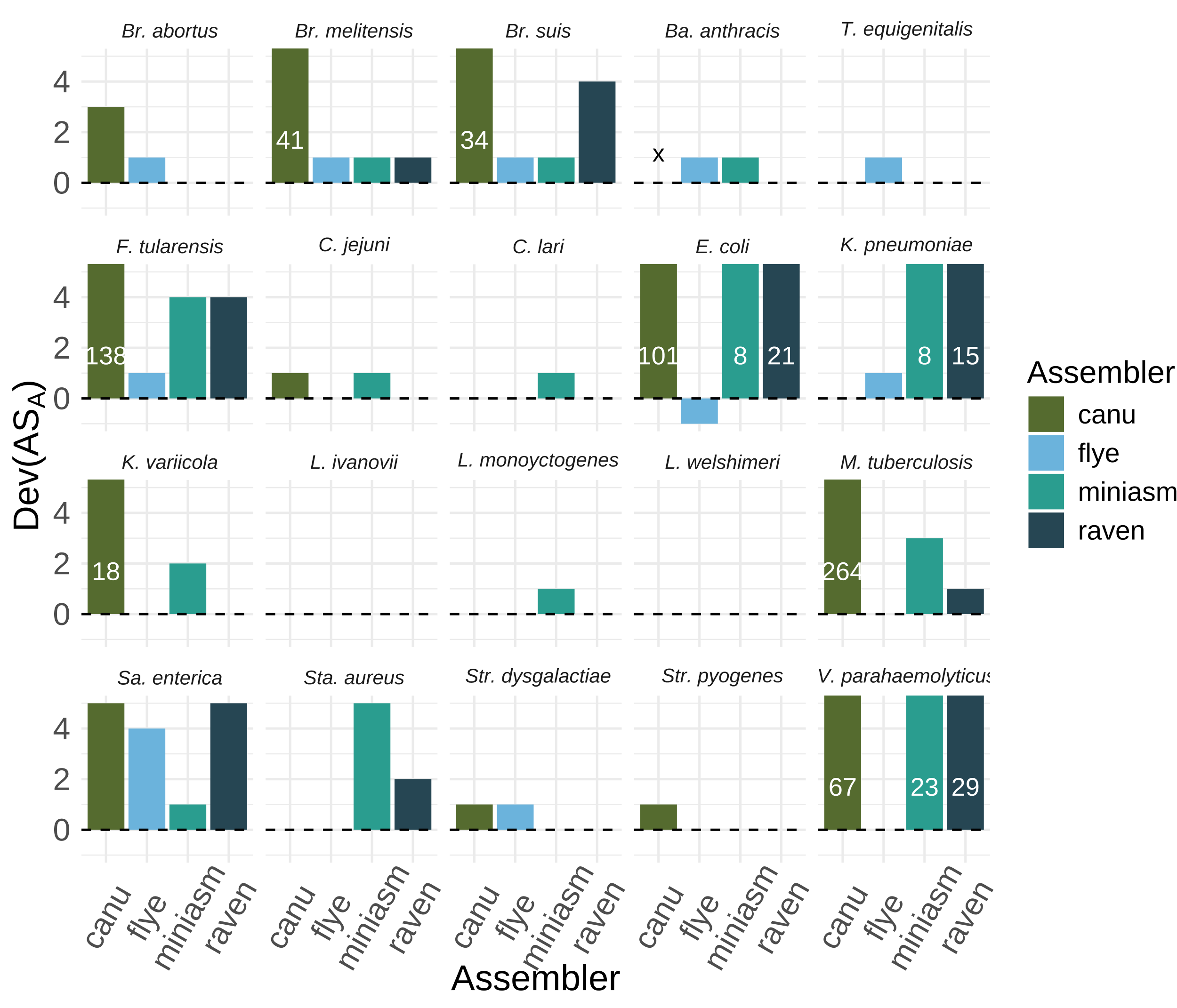


**Figure S2:** Continuity of final assemblies for each assembler (all strains). The deviation $Dev\left( AS_{A} \right)$ (y-axis) for each strain and assembler. The white numbers indicate $Dev\left( AS_{A} \right)$, when $Dev\left( AS_{A} \right)>5$. An “X” indicates that the assembly process failed. The colors indicate the different assemblers (on the x-axis).
